# Novel antimicrobial phosphate-free glass–ceramic scaffolds for bone tissue regeneration

**DOI:** 10.1038/s41598-020-68370-y

**Published:** 2020-08-21

**Authors:** M. Suárez, E. Fernández-García, A. Fernández, R. López-Píriz, R. Díaz, R. Torrecillas

**Affiliations:** 1 Nanomaterials and Nanotechnology Research Center (CINN-CSIC), Universidad de Oviedo (UO), Principado de Asturias, Avda de la Vega 4-6, 33940 El Entrego, Spain; 2 Instituto de Investigación Sanitaria del Principado de Asturias, Av. Roma, s/n, 33011 Oviedo, Asturias Spain

**Keywords:** Biological techniques, Cell biology, Microbiology, Health care, Materials science

## Abstract

In this study a phosphate-free glass–ceramic porous scaffold was synthesized by a three-step methodology involving slurry preparation, induction of porosity by surfactant-assisted foaming following by freeze-drying and sintering. This inorganic scaffold was characterized by X-ray diffraction, scanning electron microscope (SEM), degradation and bioactivity. Thermal treatment at 750 °C showed two new crystalline phases, combeite and nepheline, into the glassy matrix responsible for its properties. The cell response of the scaffold was also evaluated for using as a bone graft substitute. A commercial Biphasic Calcium Phosphate, BCP, scaffold was assessed in parallel as reference material. Microstructures obtained by SEM showed the presence of macro, meso and microporosity. The glass–ceramic scaffold possesses an interconnected porosity around 31% with a crack-pore system that promote the protein adsorption and cell attachment. Glass–ceramic scaffold with high concentration of calcium ions shows an antimicrobial behavior against *Escherichia coli* after 24 h of contact. Nepheline phase present in the glass–ceramic structure is responsible for its high mechanical properties being around 87 MPa. Glass–ceramic scaffold promotes greater protein adsorption and therefore the attachment, spreading and osteodifferentiation of Adipose Derived Stem Cells than BCP scaffold. A higher calcification was induced by glass–ceramic scaffold compared to reference BCP material.

## Introduction

Bone tissue regeneration remains as an important challenge in the dental field, orthopedic and craniofacial surgery^1,2^. Orthopedic implants often require an extra surgery because of presenting a lack of three of the most critical characteristics of living tissues: (1) the ability to self-repair, (2) the ability to maintain a blood supply and (3) the capacity to modify their particular structure and properties in response to the local environment^3^. Bone tissue engineering (BTE) arises to solve those problems, promoting the self-regeneration of those diseased or damaged bone tissues^4^. The cornerstone of BTE is the synergistic combination of osteogenic cells, biocompatible framework or scaffold, vascularization and specialized culture conditions with suitable biochemical and physical stimuli to ultimately promoting ingrowth and physiological restoration of bone^5-7^.

Synthetic bone graft substitutes based on a wide range of biomaterials have been developed during the last decades. In this way, inorganic scaffolds are recognized for their biocompatibility, osteoconductivity and bioresorbability^8^ and play a fundamental role in regeneration because they provide a temporary three-dimensional physical support during bone healing and interact with the biological milieu to regulate cell attachment, migration and differentiation of those cells involved in osteogenesis^9-11^. The development of scaffolds is constantly evolving, seeking to develop synthetic micro environments with potential to mimic the regulatory characteristics of natural extracellular matrices^12^.

Bioactive glasses (BGs) caused a revolution in healthcare and have been used in BTE for decades^13-15^. They provide a structural support and strong anchorage to endogenous bone^16^^,^ present a positive effect on vascularization^17^ and antimicrobial properties^18^. The latter constitutes an important topic because microbial infection is a major problem in modern medicine. The dissolution products from such glasses, mainly silicon and calcium ions, can upregulate the expression of genes that control osteogenesis^19-21^. 45S5 Bioglass discovered by Hench in 1969 is the first example of a biomaterial belonging to the third generation since it was the first material to bond with bone, rather than be encapsulated by fibrous tissue. However, 45S5 Bioglass fabricated using the replication method was brittle with low fracture toughness and compressive strength^22^. In addition to the silicate BGs, many new compositions have been proposed for optimizing the body´s response according to the specific clinical applications as borate glasses, which stand out for their high dissolution rates and apatite-forming ability^23^^,^ phosphate glasses with less bioactivity but high solubility and BGs with other cations within the glass network in order to confer additional beneficial properties^24^ and also glass–ceramic. J. Moya et al.^25-27^ had developed a new family P_2_O_5_-free glass–ceramic material in the SiO_2_-Na_2_O-CaO-B_2_O_3_ system through a sinter-crystallization process that allows the formation of combeite and nepheline phases in a residual glass matrix. This new bioactive glass–ceramic has been proposed to manufacture antimicrobial biomaterials with a wide range of clinical applications, such as bone graft substitutes^28^.

A number of methods have been used to control the porosity of a scaffold. The combination of the freeze-drying and leaching template techniques^29^^,^ supercritical CO_2_ foaming and melt processing, robocasting^30,31^, immersion-precipitation, freeze casting^32^^,^ salt leaching, laser sintering, electrospinning method and direct Melt ElectroWriting (MEW) technique^33^. However, most techniques have some drawbacks: They need special equipment, are often very expensive, involve organic/inorganic porogens, templates or complicated chemical process or technology.

This work aims to prepare for the first time porous scaffolds in the specified system developing a simple methodology that combines surfactant-assisted foaming and freeze-drying. These inorganic scaffolds are exhaustively characterized in terms of microstructure, crystalline phases, porosity, degradation, bioactivity and antimicrobial activity. Our second goal is the combination of the porous scaffolds with adult stem cells, as a feasible approach to reconstruct large bone defects^34^. Adipose-derived stem cells (ADSCs) are selected by being very similar to marrow-derived stem cells (MDSCs) but easier to harvest, with minimally invasive procedure, and in a large amount for tissue-engineering applications^35^. Furthermore, ADSCs present anti-inflammatory and immunomodulatory properties^36^. The ability to filling scaffolds with ADSCs, as well as their capability to adhere, proliferate, differentiate and produce a mineralized matrix at the new glass–ceramic constructs was evaluated. A Biphasic Calcium Phosphate (BCP) commercially registered as Repros was assessed in parallel as reference material.

## Experimental section

### Manufacturing of highly porous scaffolds

In this study, a glass powder chemically composed of 40.3 SiO_2_, 8.5 B_2_O_3_, 18.8 Na_2_O, 19.2 CaO, 0.57 K_2_O, 11.7 Al_2_O_3_, 0.12 Fe_2_O_3_ and 0.81 of minor oxides (wt%), with a particle size of d_50_ = 13.05 ± 0.1 µm determined by using a particle size analyzer (LS Particle Size Analyzer 13 320, Beckman Coulter, Indianapolis, IN, USA) was used as starting material. The scaffolds were fabricated using a three-step methodology involving slurry preparation, induction of porosity by a surfactant-assisted foaming, freeze-drying and sintering of porous green scaffolds. For this, scaffolds with 60 and 70 wt% of solid content were prepared by adding a percentage (0.2–0.4 wt%) of surfactant agent (Tritón X 100, Panreac, Spain) and 10 wt% of binder (Duramax B1000, Rohm & Haas, USA) to 30 g of the slurry. Air bubbles were incorporated by continuous stirring with a mechanical shaker for 5 min. The homogeneous glass slurries incorporating uniformly-distributed bubbles throughout their volume were freeze-dried, thus obtaining green scaffolds. The treatment consisted of freezing at -19 °C for 24 h, followed by extraction of the ice crystals in a lyophilizer (CryoDos, Telstar, Spain) under vacuum (0.1 mbar) and held to -50 °C for 72 h. Glass–ceramic scaffolds were obtained by sintering at 750 °C for 1 h with a heating rate of 5 °C·min^-1^.The reference material Repros (BCP) (JRI Orthopaedics Ltd., Sheffield, UK) is composed of 60% hydroxyapatite and 40% β-tricalcium phosphate.

### Physicochemical characterization of the scaffolds

#### Porosity

The apparent density of the studied scaffolds was determined by using the Archimedes principle. Previously, 30 min vacuum treatment was used to remove the air inside of pores. Then the penetration of water inside the pores of the scaffold was forced. After this, the apparent porosity was calculated using the Eq. (1).

1 $$AP\% = \frac{{w_{2} - w_{1} }}{{w_{2} - w_{3} }} 100$$

where w_2_ is the mass of the saturated sample weighed in air, w_1_ is the mass of the dried sample and w_3_ is the apparent mass of the saturated sample weighed in liquid.

#### X-ray diffraction (XRD)

Phases and crystallinity of scaffolds were characterized by X-ray diffraction (XRD, AXS D8 Advance, Bruker, UK), by using Cu-Kα radiation (λ = 0.15406 nm) in the range from 5° to 70°. The step was 0.02° and the step time 0.5 s. The measurement conditions used were copper anticathode water cooled with an intensity of 40 mA and a current of 30 kV. The identification of the crystalline phases was realized by using diffraction pattern files provided by JCPDS (International Centre for Diffraction Data).

#### Degradation and bioactivity test

The assessment of in vitro degradation of scaffolds was carried out by immersing 0.05 g of scaffold in 10 mL of buffer solution (Tris–HCl). The buffer solution was prepared according to EN ISO 10,993–14:2001. The Tris–HCl solution was prepared by dissolving tris-hydroxymethylaminomethane (ACS reagent, ≥ 99.8%, Sigma-Aldrich, Spain) in water (grade 2) with buffering at pH 7.4 ± 0.1 by 1 mol/L hydrochloric acid (ACS reagent, 37%, Sigma-Aldrich, Spain) at 37 °C. The bioactivity of the samples was studied in simulated body fluid (SBF) at 37 °C, 120 rpm by immersing 0.05 g of scaffold in 10 mL of SBF. Simulated body fluid (SBF) was prepared as described by Kokubo and Takadama^37^. For degradation and bioactivity test six samples were collected after 2, 6, 72, 168, 336 and 672 h soaking at 37 °C and 120 rpm, respectively. At each time point, the samples were taken out, rinsed with deionized water and dried in an oven at 120 °C for 24 h. The element concentrations in the immersion solution at different time were analyzed by inductively coupled plasma-mass spectroscopy (ICP-MS 7700x, Agilent, US).

#### Morphology

The microstructural characterization of the scaffolds was performed by Scanning Electron Microscopy (SEM, Hitachi TM 3000, Japan). The pore size and its distribution are also calculated from SEM images using ImageJ software (ImageJ, U.S. National Institutes of Health, Bethesda, Maryland, USA). Chemical composition was analyzed by Elemental Dispersive Spectroscopy (EDS, Quantax 70).

#### Mechanical properties

Uniaxial compression testing was conducted to investigate the mechanical properties of glass–ceramic and Repros (BCP) scaffolds by using a universal mechanical testing machine (Shimadzu-Serie AGS-IX, Japan). Briefly, the scaffolds with nominal dimensions: 10 × 10 × 10 mm^3^ were fixed on the testing platen. The compressive strength was determined from the maximum load of the obtained stress–strain curve obtained from the load–displacement measurements using a 10 kN load cell at a crosshead speed of 0.5 mm/min. The stress (σ) was evaluated using the Eq. (2). Ten samples (n = 10) were used for each scaffold type and data were presented as mean ± standard deviation (SD).

2 $$\sigma = \frac{4F}{{d^{2} }}$$

where F is the load under the compressive test and d is the length of the scaffold.

### Biological interactions in vitro

#### Antimicrobial activity of Glass–ceramic scaffolds

The potential of the new glass–ceramic scaffolds to inhibit the growth of both gram-negative and gram-positive strains is assessed by using *Escherichia coli* DH10B (ATCC 207151) and *Staphylococcus aureus* (ATCC 25923) as model microorganisms. Two additional tests are conducted to evaluate the antibacterial activity due to direct contact and/or to dissolution products. Bacteria are grown in Luria Bertani (LB) broth (Difco, BD Diagnostics, Sparks, MD, USA) up to approx. 1.0 × 10^6^ CFU ml^−1^. On one side, an inoculum of bacterial suspension (5 µl) is pipetted into the scaffolds (n = 3), and samples incubated at 37 °C in a humid atmosphere for 24 h. Microorganisms are then extracted from the inorganic constructs by shaking in phosphate-buffered saline solution (PBS, Sigma-Aldrich, Spain) (10 ml).On the other side, scaffolds (n = 3) are immersed into the bacterial suspension (200 µl), and incubated in agitation at 37 °C for 24 h. In both experiments, the number of survivors is determined by serial dilution plating.

#### Protein adsorption

The adsorption of model proteins on scaffold specimens was compared. Sterilized scaffolds (n = 9) were incubated in PBS (10 mM, pH 7.4) containing bovine serum albumin (BSA; 350 μg ml^−1^) or fetal bovine serum (FBS; 1 vol.%) at 37 °C for 60 min^38^. After incubation, the scaffolds were PBS-rinsed twice and the proteins extracted with a 2.0 wt% sodium dodecylsulfate (SDS, Sigma Aldrich, Spain) solution for 24 h. The total protein concentration was quantified with a microplate reader (BIO-RAD, Model 680, US) at λ = 530 nm with a commercial protein assay kit (BCA, Thermo Scientific Pierce, Spain) following the manufacturer’s instructions. Protein quantities are reported as μg of proteins per mm^3^ of scaffold measured from a BSA calibration standard (BSA, Thermo Scientific Pierce, Spain).

### ADSC response to ceramic scaffolds

#### Ethics statements

Adipose Derived Stem Cells (ADSCs) were obtained by the Central University Hospital of Asturias (HUCA, Spain) from discarded tissues after a biopsy by ethically approved protocols. Informed consent was waved by Internal Review board**.** All methods were performed in compliance with the Declaration of Helsinki.

#### Cell culture

ADSCs are harvested from omentum of informed donors during surgery at the Central University Hospital of Asturias (HUCA, Spain). Cellular isolating is made using high-glucose DMEM-Dulbecco's Modified Eagle Medium (GIBCO, Thermo Scientific, Spain) with 10% fetal bovine serum (FBS, PAA Laboratories GmbH, Austria) and 1% antibiotics (HyClone, 10 U mL^−1^ of penicillin and 10 mg mL^−1^ of streptomycin). Non-adherent cells are removed by medium exchange after 24 h and, afterwards every 2–3 days. Cells are maintained to 70–80% confluence at 37 °C in a balanced 5% CO_2_ atmosphere. Biological assays are carried out with cells at fourth passage in order to avoid old-age signs.

#### Cell adhesion and proliferation

Cellular attachment was analyzed by SEM (Hitachi TM 3000, Japan) 24 and 48 h after seeding (104 cells ml^-1^). At each study time, those ADSCs adhered at scaffolds were fixed with 2.5% paraformaldehyde (PFA, Sigma Aldrich, Spain), dehydrated within an Et-OH gradient, and dried in a chamber with silica gel.

Cell proliferation was evaluated 3 and 7 days after plating (104 cells ml^−1^) by measuring the cytoplasmic lactate dehydrogenase (LDH) activity with the Cytotoxicity Detection Kit^PLUS^ (Roche Diagnostics, Merck, Spain) as per manufacturer’s instructions. Protein extraction was made with the Mammalian Protein Extraction Reagent (M-PER, Thermo Scientific, Spain). The reduction of tetrazolium salts into formazan dye, a reaction coupled to LDH activity, was spectrophotometrically measured at 492 nm using a microplate reader (BIO-RAD, Model 680, US). The greater the absorbance value, the greater the number of proliferating cells into the scaffold. Results were normalized with the corresponding value of total protein determined with the Piece BCA Protein Assay Kit (BCA, Thermo Scientific Pierce, Spain). The percent cell viability was calculated according to Eq. (3):

3 $$\% Viability = 100 \times \frac{{Abs_{sample} }}{{Abs_{blank} }}$$

#### Osteoblastic differentiation and mineralization

Osteoblastic differentiation starts with a matrix maturation phase that is characterized by a maximal expression of certain markers, such as alkaline phosphatase (ALP). This was quantified 7 and 14 days after seeding (104 cells ml^-1^) with the SensoLyte pNPP Alkaline Phosphatase Assay Kit (AnaSpec Inc.). Protein extraction was made with the Mammalian Protein Extraction Reagent (M-PER, Thermo Scientific, Spain). Cellular extracts were incubated for 15 min at room temperature with the colorimetric substrate, pNPP, and then spectrophotometrically evaluated at 492 nm with microplate reader (BIO-RAD, Model 680, US). Results were also normalized with the corresponding value of total protein determined with the Piece BCA Protein Assay Kit (BCA, Thermo Scientific Pierce, Spain).

Alizarin Red Solution (ARS, Merck, Germany) staining is used to evaluate extracellular calcium-rich deposits in the investigated scaffolds after 21 days, which are an indication of in vitro bone formation. After 21 days in cell culture, specimens were PBS-rinsed with, fixed in cold Et-OH (70% v/v) for 1 h, and then stained for 10–12 min with 2% ARS solution prepared as indicated by Gregory et al.^39^. The unincorporated dye was removed with distilled water. The calcium deposits are specifically stained bright orange-red with ARS staining.

Osteogenic factors (50 μg ml^-1^ of ascorbic acid, 10 mM β-glycerol phosphate and 100 mM dexamethasone (all reagents from Sigma-Aldrich, USA) were added from the third day onwards.

## Results

### Selection of the optimal scaffold

The porosity of the synthetized scaffolds was obtained using the Eq. (1). According with the results showed in Table 1 increasing the solids loading from 60 to 70% caused a decrease in porosity from 65 to 35% approximately. In case of Repros (BCP) scaffolds, the total porosity percentage measured by Archimedes method is 65.6%.

**Table 1:** Composition of slurries and values of porosity for scaffolds synthesized.

Sample	Solid content (%)	Surfactant content (%)	Binder content (%)	Open porosity (%)	Closed porosity (%)	Total porosity (%)
ATE-G3-1	60	0.2	10	63.1	1.1	64.2
ATE-G3-2	60	0.4	10	59.6	1.7	61.2
ATE-G3-3	70	0.17	10	36.1	1.8	37.8
ATE-G3-4	70	0.36	10	28.4	2.6	31.1

According to the results shown in Table 1, as the solid content increases the green density increases and the porosity decreases in the final material. The different value of porosity between Repros (BCP) and glass–ceramic scaffolds could be attributed to both structural variations (e.g. different solid content) and manufacturing processes (e.g. different method to create porosity in the material, sintering temperatures and conditions, shrinkage after sintering). Taking account the values of compressive strength in natural bone, ATE-G3-4 (labeled from now as ATE-G3) was selected as the best scaffold synthetized and it was used for the rest of characterization made.

### Mineralogical phases characterization

**Figure 1:**
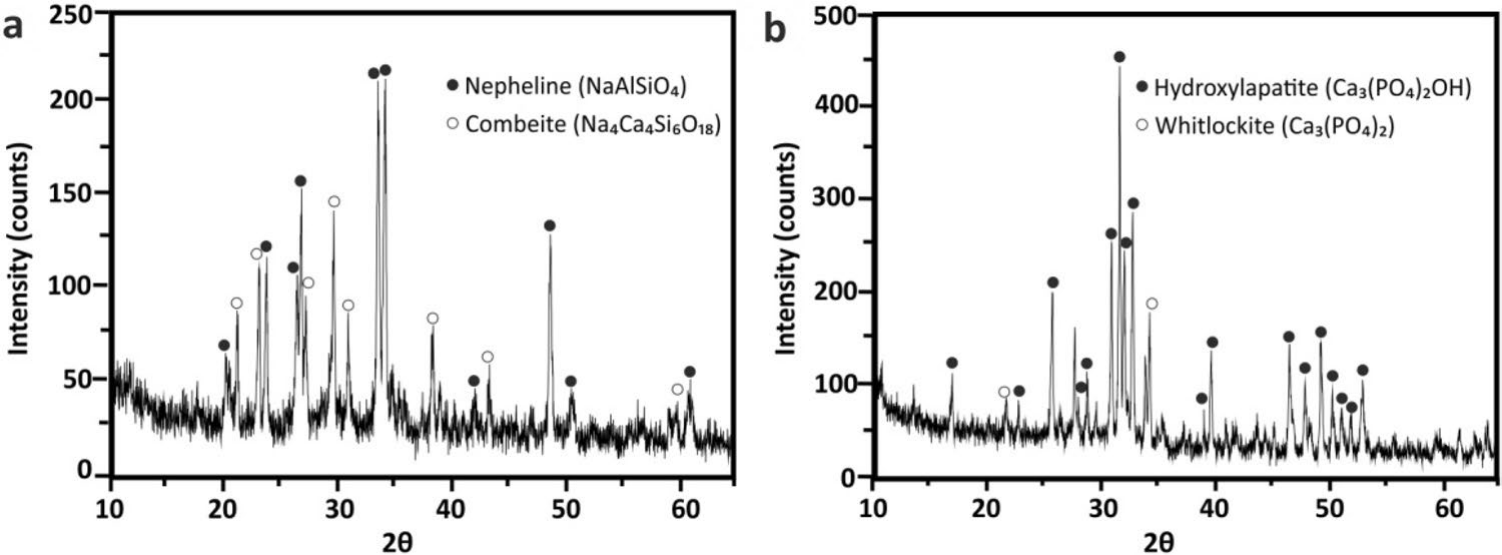
XRD pattern of ATE-G3 scaffold (**a**) and Repros (BCP) scaffold (**b**).

XRD patterns for both ATE-G3 and Repros (BCP) scaffolds are shown in Fig. 1a and b, respectively. The XRD study conducted on ATE-G3 after a thermal treatment at 750 °C showed that devitrification of the glass lead to a glass–ceramic material composed by combeite (Na_4_Ca_4_(Si_6_O_18_), PDF 75–1687) and nepheline (NaAlSiO_4_, PDF 19–1176) coexist with the glassy matrix. In case of Repros (BCP) scaffold two crystalline phases appears corresponding to hydroxylapatite (Ca_5_(PO_4_)_3_(OH), PDF 73–8419) and whitlockite (Ca_3_(PO_4_)_2_, PDF 70–2065), evidenced the non-existence of peaks from other phases. According to Rietveld analysis the Repros (BCP) scaffold composition is 59.6% of hydroxylapatite and 40.4% of whitlockite.

### Biodegradation and bioactivity results

**Figure 2:**
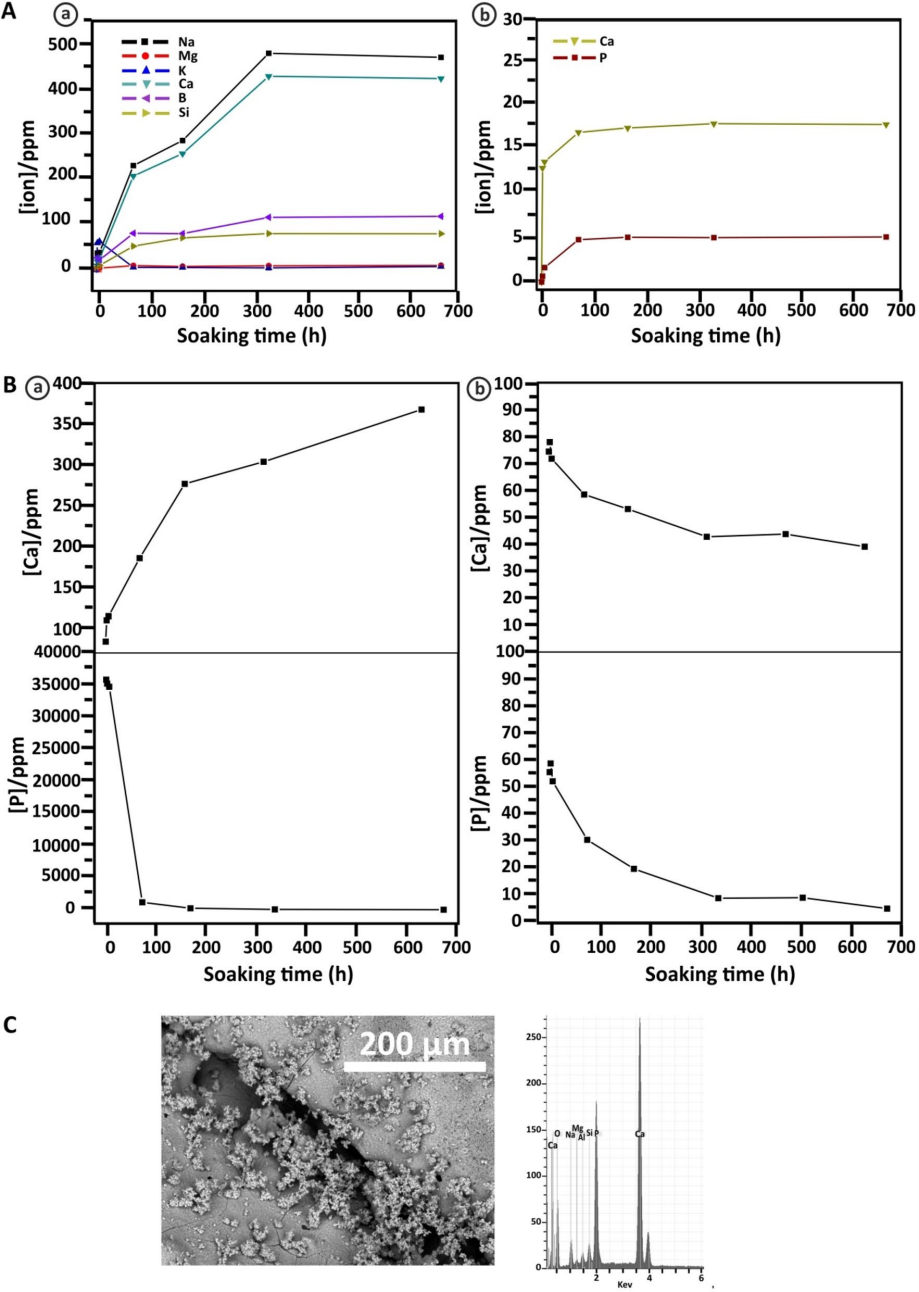
Biodegradation and bioactivity behavior of scaffolds: (**A**) Changes of the ions concentration of the ATE-G3 (**a**) and Repros (BCP) (**b**) scaffolds with soaking time in Tris–HCl (n = 3; error bars are SD). (**B**) Change of the Ca and P concentrations of the ATE-G3 (**a**) and Repros (BCP) (**b**) scaffolds with soaking time in SBF (n = 3; error bars are SD). (**C**) SEM image and EDS spectrum of mineralized ATE-G3 scaffold.

The 3D scaffolds were treated in buffer Tris–HCl for different intervals of time in order to determine the scaffold bioresorption. During the immersion in Tris–HCl solution, the scaffolds were attacked by the surrounding environment and the elements were leached from them. The concentration of leached ions at different immersing time points is determined by ICP-MS. In case of ATE-G3 scaffold (Fig. 2A (a)), the concentration of the leached ions increases as the soaking time increases indicating the degradation progress of the scaffold. The increase of the alkaline ions from the glass causes a slight increase of the pH value but it is not a detrimental for cell survival. The behavior in case of calcium-phosphate scaffolds (Fig. 2A (b)) is the same as in ATE-G3 scaffold. Increasing the soaking time, the degradation amount increases. In both cases, the percentage of degradation is high at short times and decreases at longer ones.

In case of study their bioactivity the scaffolds were incubated in SBF until 672 h. In the same way the dissolution profiles for the scaffolds in SBF solution as function of soaking time are shown in Fig. 2B a and b for ATE-G3 and Repros (BCP) scaffolds, respectively. The results show curves with different profiles. In the case of Repros (BCP) scaffolds (Fig. 2B (b)) the content of calcium and phosphate ions decreases as the soaking time increases, meaning the calcium and phosphate forming a hydroxycarbonate apatite (HCA) phase on the scaffold surface due to a process of nucleation and precipitation. However, in case of ATE-G3 scaffolds (Fig. 2B (a)) the amount of calcium increases with the soaking time but this increase is less than in case of a Tris–HCl solution. This is a compromise between the amount of calcium released from the scaffold and the amount deposited on it from the SBF solution. The concentration of phosphate decreases with the soaking time increases, showing the bioactivity behavior of the material and being it more bioactive than BCP scaffold since the amount of [PO_4_^3-^] measured in the SBF is lower in ATE-G3 compared to Repros (BCP).

The mineralization of the ATE-G3 scaffold was confirmed by SEM and EDS (Fig. 2C) after immersion for 3 days in SBF. Elemental analysis revealed a Ca/P ratio of 1.7, suggesting the presence of an apatite-like mineralized phase indicating the bioactivity of the material.

### Scaffold morphology and microstructure analysis

**Figure 3:**
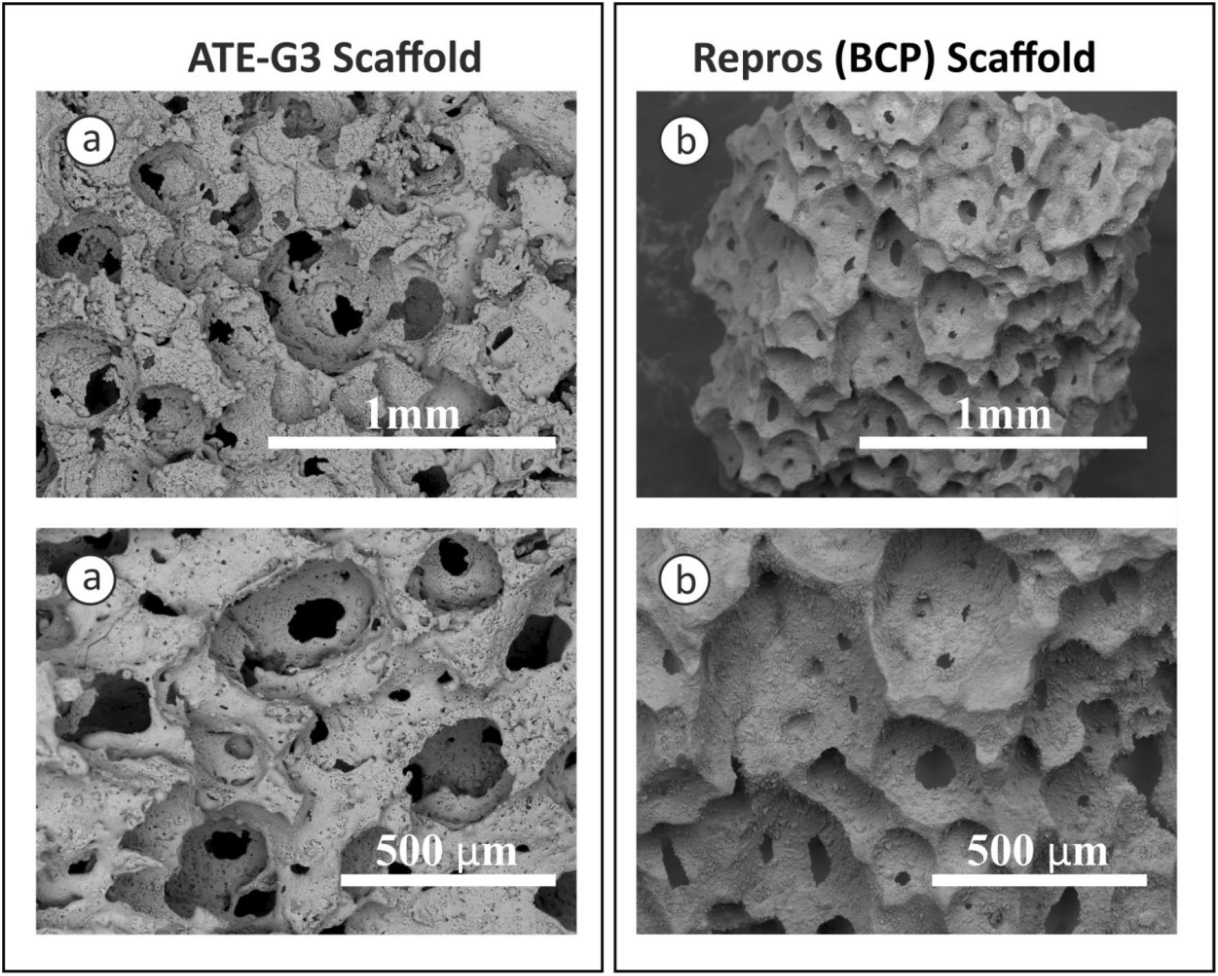
SEM micrographs for ATE-G3 scaffold (**a**) and for Repros (BCP) scaffold (**b**).

SEM micrographs of ATE-G3 glass–ceramic and Repros (BCP) scaffold, respectively are presented in Fig. 3. ATE-G3 glass–ceramic and Repros (BCP) scaffolds present macropores with pore sizes higher than 200 microns, mesopores and micropores with a pore size lower than 10 microns. The images also show that the scaffolds were well interconnected which could be used as template for cell attachment and bone extracellular matrix formation.

### Mechanical properties

Compression tests for the ATE-G3 and Repros (BCP) scaffolds were investigated by a compression testing machine. In both cases the failure process involves a fracture with multi-peak profile due to successive cracking of scaffold struts until the collapse of the structure. The compressive strength of the scaffolds calculated as the maximum compression force divided by the cross-sectional area of tested specimen, was 87.4 ± 22.1 and 6.5 ± 0.8 MPa for ATE-G3 and Repros (BCP) scaffolds, respectively. The compressive strength of glass–ceramic scaffolds is more than thirteen times higher than this value in case of Repros (BCP) scaffolds.

### Biological response

#### Antimicrobial properties

**Figure 4:**
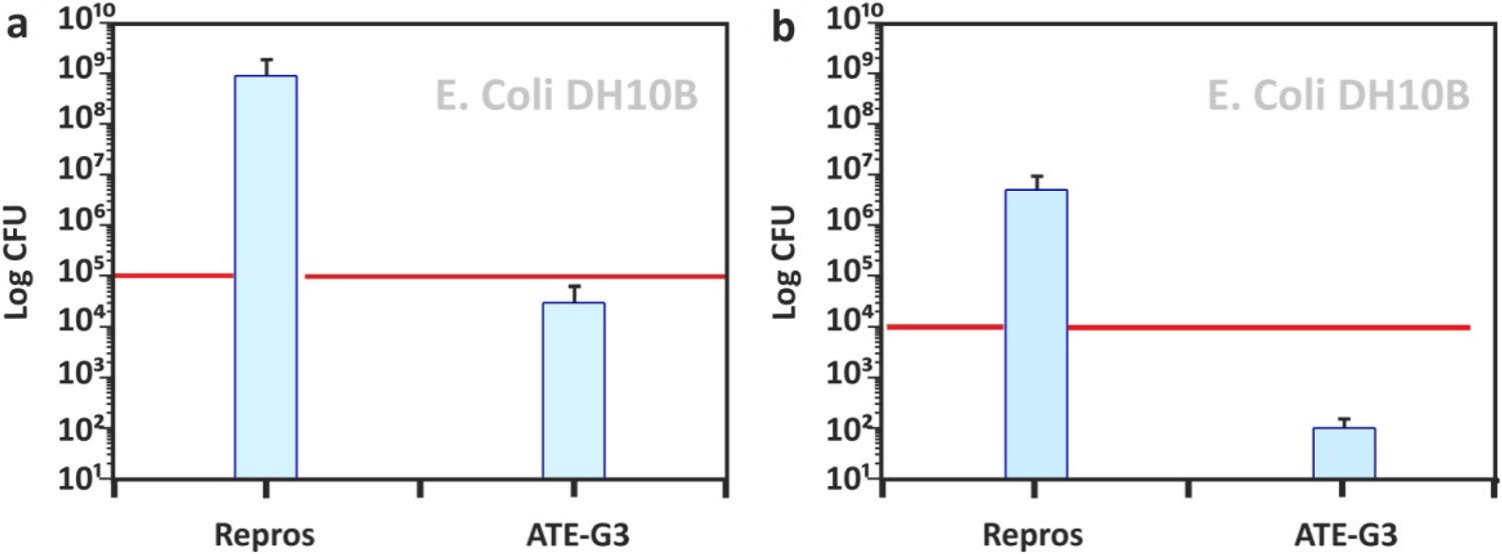
Antimicrobial activity of high porous ATEG3 compared to Repros (BCP) scaffolds in indirect contact (**a**) and direct contact (**b**). In each graph, the red line denotes the initial microorganism count. Log 10^2^ is considered the detection limit, below which no bacterial growth is considered. CFU denotes colony-forming units.

Figure 4 presents the antimicrobial activity of ATE-G3 and Repros (BCP) scaffolds assessed by inoculating the bacterial suspension within the scaffold (indirect contact; Fig. 4a) or by incubating specimens along with the bacterial suspension in agitation (direct contact; Fig. 4b). The latter allows knowing the effect of dissolution products released from the scaffolds on bacterial growth. Repros (BCP) did not interfere on the growth of *E. coli* counting higher CFU values after the incubation period. However, the interference of ATE-G3 glass–ceramic scaffolds on bacterial growth was manifested 24 h after contacting. At that point, a 5-log reduction was found when bacteria were indirectly incubated within the scaffolds whereas a 4-log reduction was found when evaluating the direct effect. The combeite crystalline phase present in the ATE-G3 material is the responsible for these antimicrobial properties as reported in a previous work.^26^.

#### Protein adsorption

**Figure 5:**
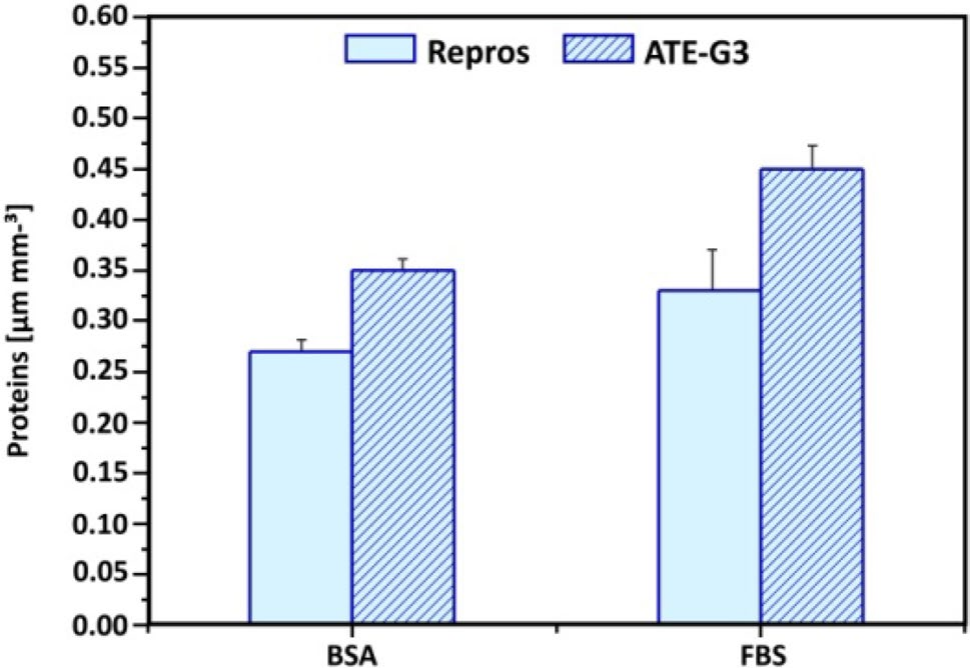
Proteins (μg·mm^−3^) adsorbed on the new ATE-G3 glass–ceramic scaffolds, when compared to Repros (BCP), after immersion in BSA (350 μg ml^−1^) and 1 vol.% FBS in PBS solutions for 60 min.

The success of the scaffold depends on its response to the cells and especially to the proteins bound to the surface of the material. In this sense the amount of proteins passively absorbed at both highly porous constructs is compared by the histograms in Fig. 5. Both ATE-G3 glass–ceramic and Repros (BCP) were able to absorb BSA and serum proteins. However, the amount of proteins adsorbed by ATE-G3 glass–ceramic was higher than by Repros (BCP). FBS proteins were significantly more adsorbed by the scaffolds than BSA protein.

#### ADSCs response to scaffolds

**Figure 6:**
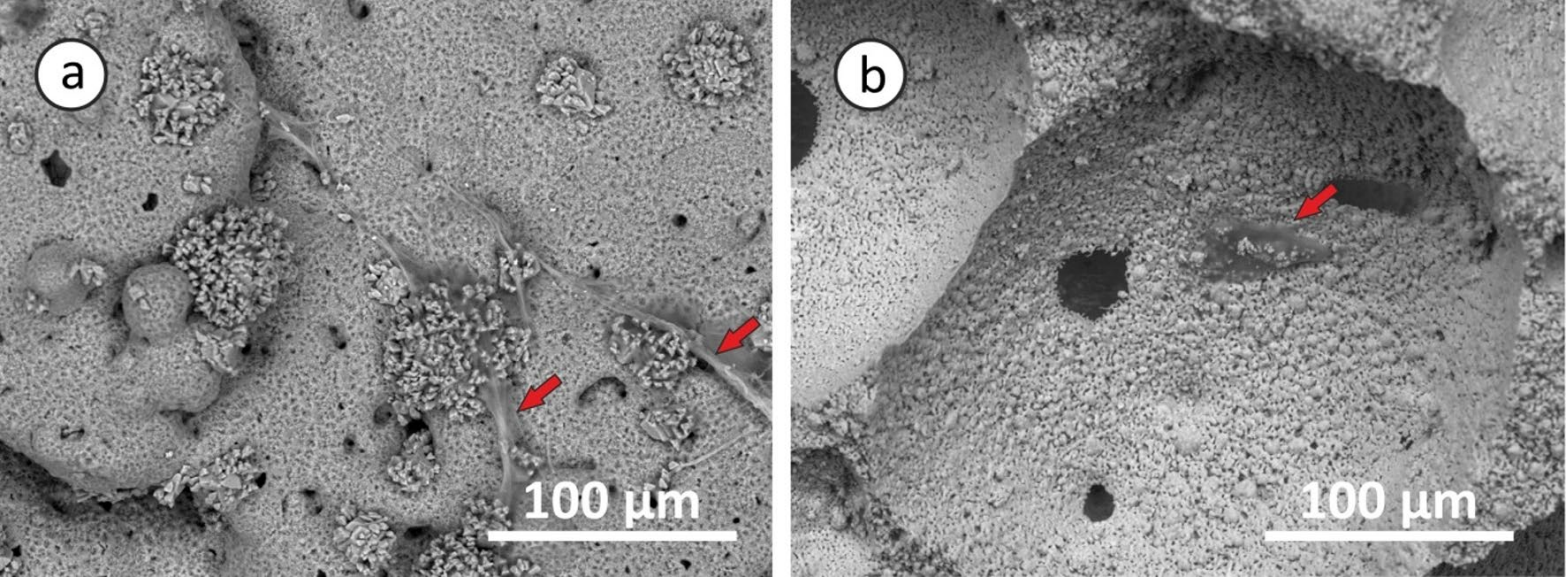
SEM images of ADSCs expanding in ATE-G3 (**a**) and Repros (BCP) (**b**) scaffolds after 24 h of seeding. Red arrows represent ADSCs cells attached on the scaffolds surface.

**Figure 7:**
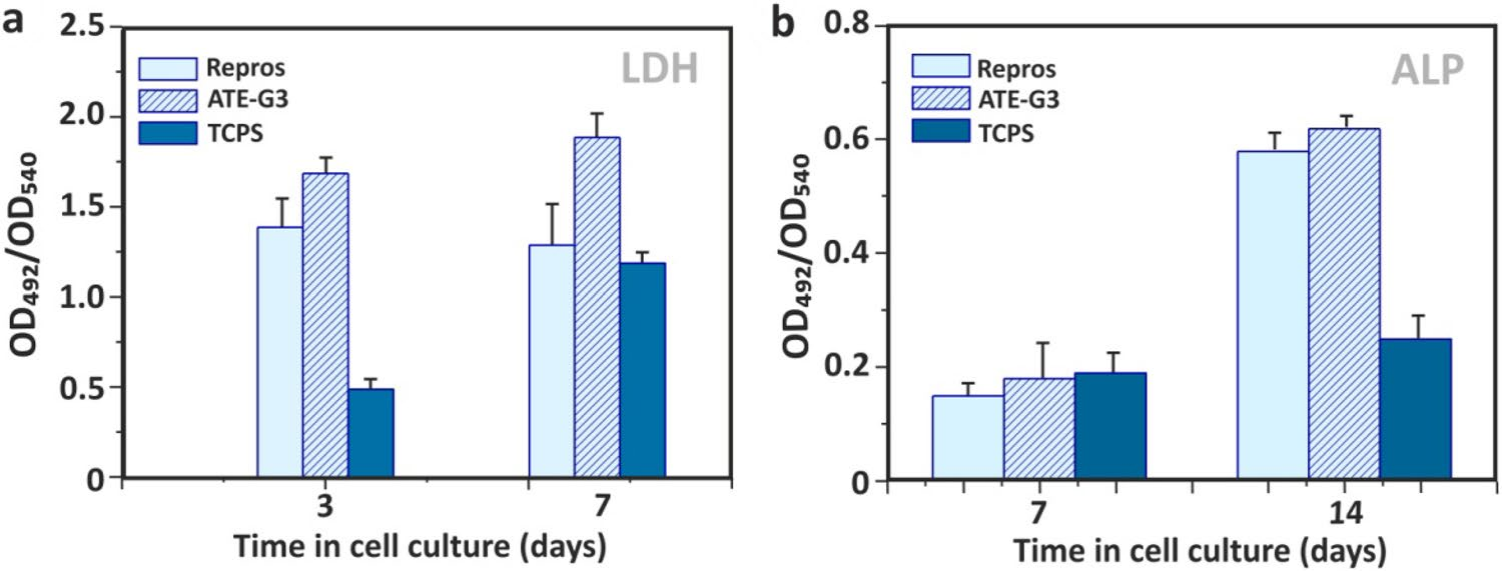
Biological response of scaffolds**.** Time course study of proliferation (LDH) and osteogenic differentiation (ALP) of ADSCs in ATE-G3 (**a**) and Repros (BCP) (**b**) scaffolds. Data are mean ± SEM values (n = 3). At each incubation time, symbols indicates statistically significantly differences, *p* < 0.05 (t-student Test).

Osteogenic differentiation of ADSCs was quantified by monitoring alkaline phosphatase (ALP) activity, calcium deposition and cell number. Cellular viability is satisfactory on the two materials since they are non-cytotoxic and able to support attachment, proliferation and viability of ADSCs. Expansion of stem cells in porous scaffolds of glass–ceramic (ATE-G3) and Repros (BCP) materials is studied by SEM. Figure 6 presents micrographs of the adult stem cells attached at the surfaces of ATE-G3 (Fig. 6a) and Repros (BCP) (Fig. 6b) scaffolds. ATE-G3 scaffolds facilitated the attachment and spreading of ADSCs. This was disclosed by an advanced adhesion, i.e. more extended cells that establish intercellular connections through long filopodia. Moreover, isolated and smaller-sized ADSCs were found on Repros (BCP) scaffolds. On the other hand, these observations correlate perfectly with the evolution of the cell number since this number in ATE-G3 glass–ceramic scaffolds is higher compared to Repros (BCP) according to the results shown in Fig. 7A (a) related to the LDH assay. ATE-G3 specimens were able to support a higher cell number than Repros (BCP) at both tested times; this difference was statistically significantly (*p* < 0.05) at 7 days where Tissue Culture Polystyrene (TCPS) was used as 2D reference.

Characterization of osteodifferentiation was performed by the measurement of ALP activity and the staining of Ca^2+^ deposits using Alizarin Red. Osteogenic factors were added from the third day in cell culture in order to mediate the differentiation of ADSCs toward the osteogenic lineage. Figure 7(b) shows the ALP enzymatic activity measured over time, indicating that this value increased statistically (*p* < 0.05) on both highly porous scaffolds along the experiment (comparison of values at 7–14 days for each scaffold) when active bone formation (osseous differentiation) occurs. Moreover, similar values of ALP were found on plain TCPS at 7 and 14 days. However, at longer culture time a clear increase in the ALP activity was observed in cells cultured on ATE-G3 glass–ceramic scaffold compared with those cultures on Repros (BCP) scaffold. These results indicate that ATE-G3 scaffold have an osteoinductive effect on the ADSCs showing a greater calcification in case of glass–ceramic scaffolds.

## Discussion

To ensure the formation of new bone, a scaffold should have one or more of these properties: osteoinduction, osteoconduction, porosity, biodegradability, mechanical stability and fabrication capability^14,40^. It is well known that the solid content has an influence on the porosity and mechanical strength and both properties are really important for scaffold materials. Porosity is necessary for the in vivo bone tissue ingrowth since it allows migration and proliferation of osteoblast and mesenchymal cells^41^ but it implies a reduction in mechanical properties compromising the structural integrity of the scaffold which could involve a risk for cells. In this way, a certain level of mechanical strength is required for the scaffolds to withstand a certain level of physiological loading. A balance must be established between these two factors^42^. Natural bone has an architectural design consisting of nanoscale to macroscopic dimensions, which provide it with stable mechanical properties being those between 145–200 MPa for cortical bone depending on species, age, anatomical site, etc^43^. Taking account the values of compressive strength in natural bone, the selected scaffold (ATE-G3-4, labelled as ATE-G3 in general) ensures to gather two necessary properties for an ideal bone substitute: porosity and mechanical property being this value around 87 ± 22 MPa. This value is really higher compared with the results obtained for Repros (BCP) scaffold and found values to those reported in the literature for bioglass and calcium phosphate scaffolds^44-46^. The improvement of the mechanical properties in case of ATE-G3 scaffolds could be ascribed to the nepheline phase (Fig. 1a) precipitated in a controlled manner by nucleation and crystal growth in a residual soda-lime glassy matrix after a devitrification process increasing the mechanical properties for this material and making it suitable to be used as bone graft substitute. In this sense, aligning the mechanical properties of the scaffolds with the specific tissue was an interesting strategy in order to advance in stem cell differentiation. Furthermore, ATE-G3 glass–ceramic scaffold with varied shapes and sizes could be successfully fabricated to be applied in treating different bone defects in contrast to 45S5 Bioglass by Hench whose poor workability is its main drawback^47^.

A scaffold is defined as a porous matrix developed to provide a defined microenvironment that promote tissue repair and regeneration since it serves as temporary structure that is replaced by native tissue subsequently so it need to be gradually removed from the implant site by a process commonly referred to as biodegradation^48^. In this sense controlled scaffold degradation is also essential characteristic for a scaffold to achieve; if a scaffold degrades too quickly, mechanical failure could occur. In this way, comparing the results showed in Fig. 2A (a) and (b) for ATE-G3 and Repros (BCP) scaffolds respectively, the trend seems the same. There is greater ion release during the early incubation times and stability at higher times. However, some differences could be observed between both materials showing a great influence on their behavior and properties. The first difference is that the amount of calcium release in ATE-G3 scaffold is higher than in Repros (BCP) and two reasons could explain this result: 1) the different chemical composition of the scaffolds. Repros (BCP) is composed by hydroxylapatite and tricalcium phosphate. Hydroxylapatite is one of the main components of bone tissue and it is bioactive but not bioresorbable and is most thermodynamically stable at a physiological pH^49^^,^ which is generally an undesirable feature for tissue engineering scaffold materials. According to the literature^50^ HA and β-TCP scaffolds are characterized by more bland surface reactions and minimal ionic release compared with bioactive glasses and 2) the degree of crystallinity since high crystallinity means low degradation. Hench et al.^51^ showed that the crystalline of the material has an influence on the apatite deposition and explained that the onset time for apatite formation increased with percentage crystallinity up to 60%. In this way Repros (BCP) scaffolds is complete crystalline while ATE-G3 is a glass–ceramic material which is partially crystallized. The second difference is that glass–ceramic scaffold release critical concentrations of soluble ions such as Si, Ca, Na which induce intracellular and extracellular responses promoting rapid bone formation. These results are related to the capacity of the material to interact with and bind to host tissue defined as bioactivity. Repros (BCP) and ATE-G3 scaffolds fulfill this property being this behavior higher in case of glass–ceramic scaffold since the amount of [PO_4_^3-^] measured in the SBF is lower compared to Repros(BCP) (see Fig. 2B (a) and (b)). In case of Repros (BCP), it is believed that the release of calcium and phosphate ions creates more precipitation of Ca-P due to increase of the ionic concentration. In ATE-G3 scaffold based on phosphate-free glass–ceramic, sodium ions leach out of the matrix and produce the dissolution of the SiO_2_ networks, forming silanol groups which gradually precipite into a silica layer where calcium and phosphate ions are deposited on producing an apatite layer on its surface^52^. Furthermore, it is believed that the presence of silicon in the material helps to the Ca^2+^ and PO_4_^3-^ deposition and consequently the rapid bone growth compared with materials with silicon-deficient environment. This could explain the bioactivity of ATE-G3 scaffold obtaining an apatite-like mineralized layer on its structure with a Ca/P ratio of 1.7 after three days of immersion in SBF.

The microstructural characteristics of the scaffolds have a strong effect on cell-material interactions^53,54^. It is well known that the interconnected porous scaffold is important for cell migration, an effective in vivo bone ingrowth and a proper vascularization. According to Hulbert et al.^41^ macropores around 100 microns are required for the penetration of cellular and extracellular components of bone and blood and pore diameters higher than 200 microns are appropriate for osteoconduction process. In our case, SEM images of the sintered scaffolds (Fig. 3 a and b) prove the presence of macro, meso and microporosity with an interconected porosity. However, slight differences are observed in case of ATE-G3 scaffold since a cracks-pores system, forming during the sintering process, is observed in its microstructure as it is shown in previous works with a dense compact of this material^55^. Chemical and physical properties of biocompatible scaffolds (e.g. chemical composition, microstructure, mechanical or electrical properties, etc.) must be taken into careful consideration since they can affect the protein adsorption and the proliferation and differentiation of stem cells^56-58^. In this sense differences between ATE-G3 glass–ceramic and Repros (BCP) scaffolds should be appointed; (1) the cracks-pores system observed in case of ATE-G3 scaffolds is a novelty compared to already existing in the literature and increases the protein adsorption since it could induce a capillary force that enhances their diffusion and migration compared with Repros (BCP) scaffolds (Fig. 5), (2) The different chemical nature of two scaffolds can lead to different charge on the substrate surface affecting to the protein adsorption. The proteins can be divided into two types generally, one is acidic proteins and the other is basic proteins. According to the literature^59^^,^ it is reported that BCP materials have negative surface charge and preferred to adsorb more basic protein than acidic protein such as BSA. In case of ATE-G3 scaffolds, the basic pH reached in the solution due to the ions released is appropriate for proteins adsorption mediating cellular attachment, and determine the subsequent osteoconduction and osteoinduction processes. In this way, the more adsorption sites the more proteins to be adsorbed on the scaffolds and they can subsequently stimulate the osteogenic-related functions of cells, such as attachment, proliferation, osteogenic differentiation and mineralization. This is related to SEM micrographs for glass–ceramic scaffolds (Fig. 6a) where more extended cells with intercellular connections through long filopodia can be observed in contrast to the isolated cells found in Repros (BCP) materials. Glass–ceramic scaffolds appear to significantly enhance human ADSC LDH and ALP activity compared with Repros (BCP) constructs indicating a higher proliferation and cell osteogenic differentiation towards the osteogenic lineage compared to Repros (BCP) scaffolds as well as other bioglass scaffolds reported in the literature^21,60^. After 5 weeks in vitro culture, Alizarin red staining revealed that extensive calcification was produced in glass–ceramic scaffolds compared to Repros (BCP) scaffolds.

Bacterial infection during or following implantation of an artificial scaffold is a serious problem since it increases morbidity and costs to national health systems. Different antibiotics have been proposed, over the years, to eradicate the infection problems. However, the use of these medications has serious consequences, such as resistance of bacteria and damage to tissues^61^. In the case of glass–ceramic scaffold the interference on bacterial growth was manifested 24 h after contacting compared to Repros (BCP) scaffolds that shows higher CFU values after the same period of incubation (Fig. 4 a and b). According to Moya et al.^25^ the anbimicrobial activity of the glass–ceramic arises from the capability to release calcium ions from the surface of the glass particle at the glass-membrane interface increasing this concentration in the proximity of the plasma membrane, which leads to a fast membrane depolarization, a blockage of electrochemical gradient restoration process in bacteria and, finally, death of the cell. Calcium is contained in both combeite crystals and the residual glassy phase of the glass–ceramic scaffold and both phases are somehow involved in its antibacterial capability, as well reported in previous work^26^. This bactericide behavior is not observed in case of Repros (BCP) scaffold.

Phosphate-free glass–ceramic scaffold is a novel material for using as bone graft substitutes since it has several advantages: simple methodology for synthesis, antimicrobial properties due to the calcium content present into its chemical composition, high bioactivity, high mechanical properties due to the crystallization of nepheline phase, and an excellent protein adsorption, attachment, proliferation and differentiation of ADSCs cells due to the cracks-pores system forming during the structure of glass–ceramic and its chemical nature.

## Conclusions

In this study a novel phosphate-free glass–ceramic scaffold was obtained by a simple method for using as three-dimensional matrix in the new bone formation. Thermal treatment at 750 °C showed that devitrification of the glass with two crystalline phase composed by combeite and nepheline that were responsible for its antimicrobial and mechanical properties. The material combines two main properties for a bone graft substitute: porosity and structural integrity with mechanical strength close to the natural bone due to the nepheline phase present in its structure. The chemical composition phosphate free of the synthetized scaffold allows a high bioactivity producing an apatite-like mineralized layer on its surface with a Ca/P ratio of 1.7 after three days of immersion in SBF. The high concentration of calcium ions explains the antimicrobial behavior of the glass–ceramic scaffold against *E. Coli* after 24 h of contact. The crack-pore system present in glass–ceramic scaffold and its chemical nature promotes greater protein adsorption and therefore the attachment, spreading and osteodifferentiation of ADSCs. A higher osteoinductive effect on the ADSCs and greater calcification was induced by ATE-G3 scaffolds compared to commercial Repros (BCP) material.
