# Author Correction: Novel antimicrobial phosphate-free glass–ceramic scaffolds for bone tissue regeneration

**DOI:** 10.1038/s41598-021-88995-x

**Published:** 2021-04-28

**Authors:** M. Suárez, E. Fernández-García, A. Fernández, R. López-Píriz, R. Díaz, R. Torrecillas

**Affiliations:** 1 Nanomaterials and Nanotechnology Research Center (CINN‑CSIC), Universidad de Oviedo (UO), Principado de Asturias, Avda de la Vega 4‑6, 33940 El Entrego, Spain; 2 Instituto de Investigación Sanitaria del Principado de Asturias, Av. Roma, s/n, 33011 Oviedo, Asturias Spain

Correction to: *Scientific Reports* 10.1038/s41598-020-68370-y, published online 21 August 2020

This Article contains a typographical error in the Results section under subheading ‘Mechanical properties’ where,

“The compressive strength of the scaffolds calculated as the maximum compression force divided by the cross-sectional area of tested specimen, was 87.4 ± 22.1 and 6.5 ± 0.8 MPa for ATE-G3 and Repros (BCP) scaffolds, respectively.”

should read:

“The compressive strength of the scaffolds calculated as the maximum compression force divided by the cross-sectional area of tested specimen, was 8.7 ± 2.2 and 6.5 ± 0.8 MPa for ATE-G3 and Repros (BCP) scaffolds, respectively.”

